# Hovering hummingbird wing aerodynamics during the annual cycle. II. Implications of wing feather moult

**DOI:** 10.1098/rsos.171766

**Published:** 2018-02-14

**Authors:** Yonathan Achache, Nir Sapir, Yossef Elimelech

**Affiliations:** 1 TASP—Technion Autonomous Systems Program, Technion - Israel Institute of Technology, Haifa, Israel; 2 Animal Flight Laboratory, Department of Evolutionary and Environmental Biology, University of Haifa, Haifa, Israel

**Keywords:** aerodynamics, hovering, hummingbird, animal flight, moult

## Abstract

Birds usually moult their feathers in a particular sequence which may incur aerodynamic, physiological and behavioural implications. Among birds, hummingbirds are unique species in their sustained hovering flight. Because hummingbirds frequently hover-feed, they must maintain sufficiently high flight capacities even when moulting their flight feathers. A hummingbird wing consists of 10 primary flight feathers whose absence during moult may strongly affect wing performance. Using dynamic similarity rules, we compared time-accurate aerodynamic loads and flow field measurements over several wing geometries that follow the natural feather moult sequence of *Calypte anna*, a common hummingbird species in western North America. Our results suggest a drop of more than 20% in lift production during the early stages of the moult sequence in which mid-wing flight feathers are moulted. We also found that the wing's ability to generate lift strongly depended on the morphological integrity of the outer primaries and leading-edge. These findings may explain the evolution of wing morphology and moult attributes. Specifically, the high overlap between adjacent wing feathers, especially at the wing tip, and the slow sequential replacement of the wing feathers result in a relatively small reduction in wing surface area during moult with limited aerodynamic implications. We present power and efficiency analyses for hover flight during moult under several plausible scenarios, suggesting that body mass reduction could be a compensatory mechanism that preserves the energetic costs of hover flight.

## 1. Introduction

The primary role of flight feathers is to form the surfaces over which lift is generated [1,2]. During the course of the year, these feathers are prone to wear and damage due to a variety of processes, including collisions with vegetation, friction with adjacent feathers and skin and the activity of parasites such as feather lice [3,4]. A possible implication of feather wear concerns its effects on flight performance, as was previously observed in pigeons where feather wear decreased lift production by more than 30% [5]. Similarly, Swaddle *et al.* [6] suggested that worn plumage may reduce a bird's take-off speed and manoeuvrability, hampering its escape flight performance. Because feather wear is irreversible, feathers must be replaced to ensure their functionality [3,7].

Feather damage is determined by several extrinsic (e.g. habitat type and radiation intensity) and intrinsic (e.g. feather durability) factors. Therefore, bird feather moult properties are shaped by these factors through natural selection which acts on feather-related functions such as flight performance, plumage attractiveness during mating (sexual selection) and body thermal regulation. Feather moult properties, including the sequence, timing, duration and extent of the moult process, may consequently differ in each bird species and even between different populations according to different factors such as body size, life-history properties and diet [3,7–11].

Feather moult is considered one of the most energy-demanding processes in the annual cycle of birds, and as such, it does not usually overlap with reproduction or migration which constitute other major, annual, energy-demanding processes [7,12]. During moult, reduced thermal insulation from the plumage and the energy cost of feather synthesis have been found to increase bird metabolism by 20–40% above the bird's basal metabolic rate [13]. Furthermore, due to morphological deficits that inevitably result from feather shedding during moult, birds may need to exert more power for flight during the extended periods over which feather replacement takes place [14,15]. The consequences of feather moult on bird aerodynamics are rarely described [7,14,16], and there is limited evidence regarding metabolic consequences of moult due to the smaller surface area of the wings. While both Epting [15] and Chai [17] found substantial increases in mass-specific metabolic rates of moulting hummingbirds, when considering the whole organism rather than mass-specific metabolism, the latter study does suggest that the flight metabolic rate of moulting hummingbirds is nearly constant. This is achieved through reduction of bird body mass during moult (see below). A number of studies have proposed that feather gaps in the wings and tail that are created during moult reduce lift [16,18–21], providing a plausible connection between wing surface deficits, deteriorated wing aerodynamics and elevated flight metabolism of moulting birds.

**Figure 1.**
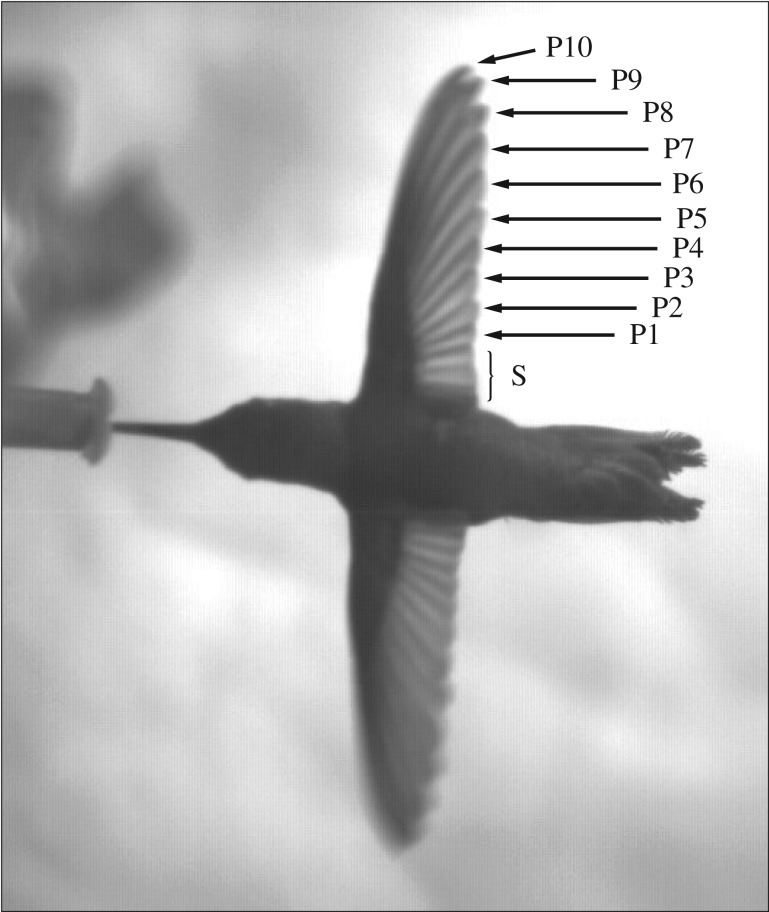
An Anna's hummingbird wing consists of 10 functional primary flight feathers denoted by P that are numbered outwards. The secondary flight feathers are denoted by S without numbering specific feathers.

We studied the consequences of wing feather moult in Anna's hummingbird (*Calypte anna*). The moult sequence of Anna's hummingbird was studied by Williamson [22] and is described as follows. The first feathers to be shed are primaries (denoted by P; figure 1) 1, 2, 3 and 4, with some variation between different individuals regarding the number of feathers that are shed simultaneously (2–4 feathers). After these feathers are fully regrown, primaries 5, 6, 7 and 8 are replaced one after the other. The secondary feathers (denoted by S; figure 1) are shed simultaneously with primary 6. The 10th primary flight feather is shed after primaries 1 through 8 are fully regrown, and P9 is shed after P10 grows to at least one-fourth of its final size. The disruption of the outwards moult sequence of the primary flight feathers, where P10 is shed before P9, is an exception from common moult sequences in nearly all other bird species [3,22,23]. We note that this moult sequence also characterizes other hummingbird species [24].

Hummingbirds are known to vary their body mass at different times of the day, notably at dusk when birds may be involved in intense feeding that could result in up to a 25% increase of their body mass [25,26]. Also, hummingbird body mass increases when the birds prepare for migration and put on weight in the form of lipids that are later used as fuel during long-distance flight [26,27]. During moult, birds were found to decrease their body mass, thereby alleviating aerodynamic forces required for weight support. Indeed, anecdotal findings, both in hummingbirds [15,17,28] and in other taxa (e.g. [29]), suggest that moulting birds reduce their body mass considerably, possibly indicating a general mechanism of mass support modifications in relation to wing surface morphology. Hummingbirds of several different species, inhabiting a wide breeding range in North America, demonstrated this pattern when examined under controlled conditions in the laboratory [15,17,28]. According to Chai [17], while wingbeat kinematic variables, such as flapping frequency and amplitude, hardly changed throughout the moulting sequence, a 25% reduction in body mass from that in other periods of the year was measured for moulting ruby-throated hummingbirds (*Archilochus colubris*). Information regarding the extent of this phenomenon during moult among free-ranging hummingbirds and the factors that govern it are still understudied. Moreover, the aerodynamic mechanisms that govern lift production and that may link wing geometries of moulting birds to reduced weight support and elevated metabolism have not yet been thoroughly investigated. Therefore, we herein explore the aerodynamic consequences of the natural sequence of flight feather moult of Anna's hummingbird using dynamically scaled hummingbird wings in fluids.

## 2. Methods and materials

**Figure 2.**
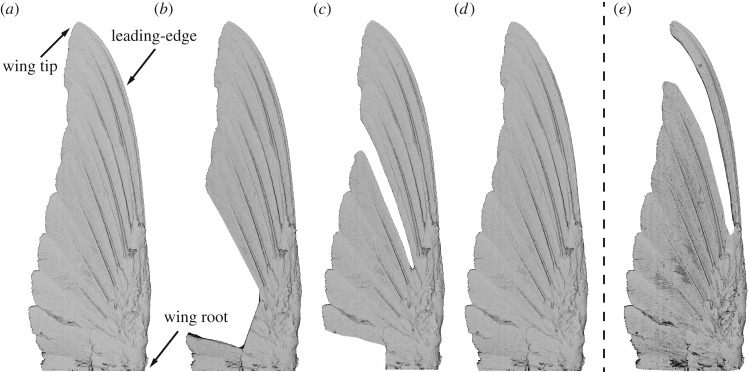
Top view of the three-dimensional printed wing geometry of moulting male Anna's hummingbird used in our experiments. (*a*) Complete wing; (*b*) P1-4 wing; (*c*) SP6 wing; (*d*) P10 wing; (*e*) P89 wing.

To study the effects of moult on the aerodynamic characteristics of Anna's hummingbird wings during hovering, time-accurate aerodynamic loads and flow field measurements were made over dynamically scaled-up model wing pairs. The models were suspended in a tank filled with a working fluid, in a propeller-like set-up, where each pair represented a different stage in the moult sequence (figure 2). The wing pairs then followed stages of initial acceleration, steady rotation and deceleration to model the downstroke of a flapping wing. The experimental set-ups have been described in detail by Achache *et al.* [30] and are briefly described below.

### 2.1. Wing design

Based on three-dimensional laser scans of a complete, dried male Anna's hummingbird wing [31], a 3.5–1 dynamically scaled wing model was fabricated (one wing span *R*=175 *mm*) using stereolithography (Objet VeroBlack^*TM*^). Similarly, following the natural moult cycle of this species [22], three representative moult stages, namely P1-4 (the shedding of primaries 1–4), SP6 (the shedding of the secondaries and primary 6) and P10 (the shedding of primary 10), were modelled by removing the corresponding flight feathers from the three-dimensional, complete wing computer-aided design model. This process was done while taking into account feather overlap (figure 2). Because the secondary flight feathers have been shown to play a minor role in lift production, their individual moult pattern was not examined in this study [17].

The wing structure of Anna's hummingbird is characterized by an increasing overlap of the primary flight feathers from the inner wing outwards, such that the feathers forming the wing tip and leading-edge largely overlap with neighbouring primary feathers (see fig. 1a in [30] or e.g. [32,33]). Because these feathers carry aerodynamic loads and are more likely to be abraded by substrates, they are prone to wear and damage. The increased overlap of the outer primary feathers may allow the bird to maintain nearly all of its wing surface even when one of the feathers is subjected to substantial damage. Furthermore, the importance of the outer primary flight feathers to flight performance is reflected by the disruption in the outward sequence of moult. All of the above suggest that wing integrity is aerodynamically critical at the leading-edge and wing tip. Therefore, we further studied the role of the wing tip in wing aerodynamics by introducing an additional wing configuration that is not part of the natural moult sequence. This was done by removing the 8th and the 9th primary flight feathers (hereafter P89 wing; figure 2*e*) in contrast to the natural moult sequence in which only one of these flight feathers is replaced at any given time.

Previous studies suggested that hummingbird wings are rigid throughout the downstroke [32,34,35]. Moreover, it was demonstrated that the porosity of the wings has very little, if any, effect on their aerodynamic performance [31,33]. The above justified the use of a rigid and non-porous, three-dimensional printed wing model to analyse Anna's hummingbird's complete wing performance and aerodynamics. To the best of our knowledge, there are no published data regarding these properties (wing deformation and air transmissivity) for a hummingbird wing during active flight feather moult. Therefore, the wing is assumed to be rigid and non-porous throughout the annual cycle for the purpose of presenting a first attempt to understand the consequences of flight feather moult on the wing's aerodynamic performance. It is understood that the results presented herein might be an overestimation of the wing's performance, which needs to be assessed by a designated study.

### 2.2. Force measurements

To measure time-accurate aerodynamic loads acting on the wing during the downstroke, the mechanical assembly was mounted on a six-component force and moment transducer (MINI40, calibration SI-20-1 by ATI Industrial Automation, Apex, NC, USA) that recorded the aerodynamic loads that acted on the wings (see fig. 1 in [30]). To match the Reynolds number of an actual Anna's hummingbird wing, as well as the transducer's effective range of measurements, the wing pair was submerged in a water–glycerin solution (*ν*=4.6 *cSt*). The time-accurate aerodynamic lift, *L*, and torque, *Q*, acting on one wing are referenced to $\frac{1}{2}\rho S{\dot{\theta }}_{\infty }^{2}R_{2}^{2}$ and $\frac{1}{2}\rho S{\dot{\theta }}_{\infty }^{2}R_{3}^{3}$, respectively. These yield the dimensionless lift and torque due to drag coefficients, *C*_*L*_ and *C*_*Q*_, where *ρ* is the fluid-specific weight and ${\dot{\theta }}_{\infty }$ is the wing's rotational rate at mid-stroke. *S*,*R*_2_ and *R*_3_ are the area and the radii of the second and third moments of area of the complete wing, respectively. The wings were tested at various angles in relation to the wing root (*α*), ranging from 5° to 35° at 5° increments. For a better signal-to-noise ratio, each experiment was repeated 50 times (*n*=50). The time-averaged aerodynamic coefficient for each individual load measurement *i* (1≤*i*≤*n*) is defined as

2.1 $${\bar{C}}_{*,i}=\frac{1}{T}\int_{0}^{T}C_{*,i}(\tau )\;d\tau ,$$

where *={*L*,*Q*}, *T* is the downstroke duration (*T*=0.74 s) and *τ* is the integration variable. Time-averaged aerodynamic coefficients are subsequently defined as

2.2 $${\bar{C}}_{*}=\frac{1}{n}\sum_{i=1}^{n}{\bar{C}}_{*,i}.$$

At the end of each wing stroke, a pause of approximately 30 s took place to establish an essentially quiescent flow at the beginning of each wing stroke.

The time-averaged lift coefficient required to support the bird's body weight (divided by two, in order to refer to one wing) is

2.3 $${\bar{C}}_{L_{b}}=\frac{m_{b}g}{\rho_{a}S_{b}{\left(2\pi f_{b}R_{2_{b}}\right)}^{2}},$$

where *m*_*b*_ is Anna's hummingbird's body mass during the moulting period, *g* is the gravitational acceleration, *ρ*_*a*_ is the air density at sea level and *f*_*b*_ is the wingbeat frequency, defined as 40 *Hz* [36]. *S*_*b*_ and *R*_2_*b*__ are the complete wing area and radius of the second moment of area, respectively [30].

### 2.3. Flow field measurements

Flow field measurements were obtained at *α*=30° using phase-locked particle image velocimetry (PIV). Our experimental set-up consisted of a PIV system (Dantec Dynamics A/S, Skovlunde, Denmark) comprising a dual-cavity 30 mJ Nd:YLF laser with a repetition rate of up to 10 Hz, a 4M pixel resolution 12 bit CCD camera (FlowSense EO) and a programmable timing unit. Two experimental schemes were applied. The first measured the in-plane velocity component at different angular positions along the downstroke (*θ*) over three span stations, namely, *z*=*r*/*R*=0.25, 0.5 and 0.75, where *r* is the dimensional distance of the span station from the wing's root. The second measured the near-wake velocity field at representative downstroke stations (see fig. 2 in [30]).

The dimensional unsteady velocity field is defined as $\text{U}=\{U,V,W\}=(1/n)\Sigma_{i=1}^{n}{\text{U}}_{i}$, where **U**_*i*_ is the flow field that was acquired at the *i*th measurement (1≤*i*≤*n*). *U*,*V* and *W* are the velocity components in the *X*,*Y* and *Z* directions, respectively (see fig. 1 in [30]). The tangential velocity at each span station is $U_{∥}(z)=\dot{\theta }R(z+\eta )$, where *η* is the dimensionless distance between the wing's root and the centre of rotation (referenced to *R*; see fig. 1d in [30]). The dimensionless velocity field in the wing's frame of reference is defined as ***u***={*U*−*U*_∥_,*V*,*W*}/*U*_∥_={*u*,*v*,*w*}. The near-wake velocity fields refer to the wing tip velocity, u*=***u***/*U*_*tip*_, where $U_{\mathrm{tip}}=\dot{\theta }R(1+\eta )$. For statistical analysis, the velocity fluctuations are defined by using the standard definition referring to the wing tip velocity at mid-stroke, $\bar{\zeta^{\prime 2}}=(1/n)\Sigma_{i=1}^{n}{\left[(\zeta_{i}-\zeta )U_{∥}/U_{\mathrm{tip}}^{90^{\circ }}\right]}^{2}$, where *ζ*=*u*,*v* or *w* (*ζ*_*i*_ denotes *ζ*'s *i*th measurement). We define a standard deviation operator of the two components of the vector field as $\sigma_{u_{1}u_{2}}={\left[\frac{1}{2}(\bar{{u^{\prime }}_{1}^{2}}+\bar{{u^{\prime }}_{2}^{2}})\right]}^{1/2}$.

## 3. Results

### 3.1. Aerodynamic loads

**Figure 3.**
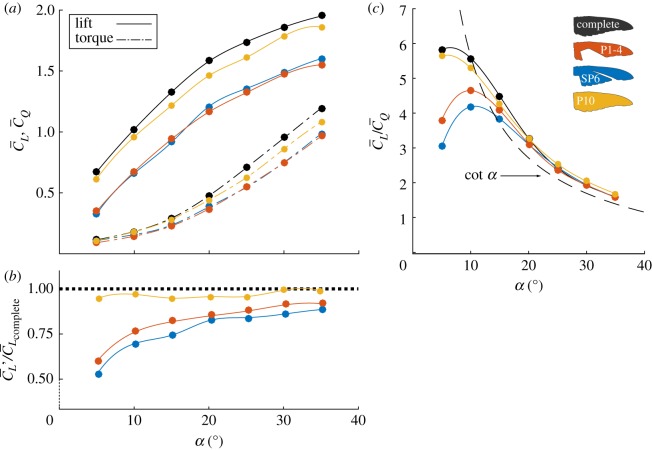
Time-averaged aerodynamic load measurements during the downstroke. (*a*) Lift and torque due to drag coefficients versus angle of attack; (*b*) ${\bar{C}}_{L}^{\prime }/{\bar{C}}_{L_{\mathrm{complete}}}$ versus angle of attack; (*c*) lift-to-torque ratio compared to the trigonometric relation for wings with an attached leading-edge vorticity, cot⁡α [37] (dashed line).

The time-averaged aerodynamic coefficients during the downstroke are presented for all tested moult stages in figure 3*a*. As expected, the complete wing produced the highest lift when compared with the wings of the three tested moult stages. At the first stage of the moult sequence, when the four innermost primary wing feathers (P1-4; figure 2*b*) have been shed, 22% of the wing surface is missing (table 1), constituting the largest area loss during the entire sequence. Lift production, at this stage, declined by $0.4{\hat{C}}_{L}$ (hat denotes unit value), compared to the complete wing for *α*>10°. In the next moult stage, when P6 and the secondary flight feathers are missing (SP6; figure 2*c*), a 10% area loss at the root and middle of the wing induced a reduction in lift capacity similar to the previous moult stage. Bridge [5] measured similar magnitudes of lift reduction over a model of a pigeon wing with gaps in the mid-wing primaries.

**Table 1.** Wing model morphology of the tested moult stages. *S* and *R*_2_ denote the wing model area and the radius of the second moment of area, respectively. Area loss is depicted by the percentage of wing surface loss in relation to the complete wing.

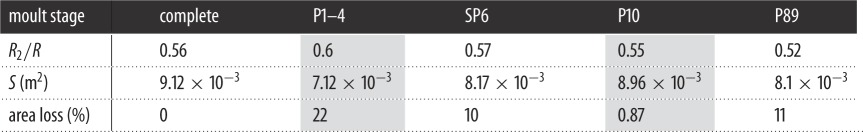

After P1-8 have fully grown in, the ascending order in which flight feathers are replaced is reversed, and P10 is shed. The rachis of P10 extends from the wing's medial section (*z*∼0.4) to the tip, and its vane forms the sharp leading-edge of the wing. Therefore, when shed, along with a 0.87% wing surface reduction (table 1 and figure 2*d*), the leading-edge thickness and shape are also altered. Because, to the best of our knowledge, the leading-edge thickness of an Anna's hummingbird wing was not measured during active moult of the P10 flight feather, in our study the thickness is a resultant of the manufacturing process as described earlier (complete, approx. 0.5 *mm*; P10, approx. 2 *mm*). In a previous study, conducted over revolving hummingbird planform wings mounted at *α*=45°, lift production was not shown to be sensitive to the change in leading-edge thickness [38]. However, over the range of tested angles of attack in this study, a reduction of $0.11{\hat{C}}_{L}$ is evident for 15°≤*α*≤25° (figure 3*a*).

In revolving wings, the tangential velocity grows linearly along the span. Therefore, loss of the medial and outer primaries would probably impair lift production more than the absence of primaries closer to the root. The moult sequence seems to follow this logic as the proximal feathers (i.e. P1-4) are shed simultaneously, opening the largest gap in the wing during the moult cycle. This is followed by a sequential replacement of the medial and outer wing feathers, where smaller gaps are opened due to both fewer feathers being shed simultaneously and the increasing overlap between adjacent feathers. To assess the effects of gap size and position in the wing on lift production, the lift coefficient for each moult stage was also calculated while taking into account each stage's corresponding wing geometry. This was done as follows:

3.1 $${\bar{C}}_{L}^{\prime }={\bar{C}}_{L}\frac{SR_{2}^{2}}{S^{\prime }R_{2}^{\prime 2}},$$

where *S*′ and *R*_2_′ are each moult stage's corresponding wing area and radius of the second moment of area, respectively (table 1). The ratio between each wing ${\bar{C}}_{L}^{\prime }$ and the complete wing lift coefficient (${\bar{C}}_{L_{\mathrm{complete}}}$) is presented in figure 3*b* and serves as a measure of wing effectiveness in terms of lift production for each moult stage. Values closer to one indicate a minor effect of the missing feathers on the wing's ability to produce lift, while lower values imply a more substantial diminished performance. The moult of the medial flight feathers (i.e. SP6) created the largest degradation in wing effectiveness (as low as 50% compared with the complete wing; figure 3*b*). The degraded performance of this moult stage is associated with a large gap opened in the wing surface with respect to the effective disc area (see fig. 5 in [30]). As most lift production is made by the medial wing section, gaps in this area cause a substantial reduction in the ability of the wing to generate lift. At a later stage of the moult sequence, namely P10, the missing surface area at the distal part of the wing (where the tangential velocity is the highest) was smaller overall, such that the wing maintained its ability to produce lift. Owing to the increasing overlap and the gradual sequence in which the medial and outer flight feathers are replaced, it is likely that if larger gaps were naturally found in the hummingbird wing as part of the regular moult sequence (e.g. through simultaneous shedding of two or three adjacent feathers), lift production would have been substantially impaired. This aspect is treated below through the introduction of the P89 wing configuration (figure 6).

Throughout the moult stages, torque values have proportionally decreased relative to the reduction in lift described above, indicating a link between lift and torque (figure 3*a*). Hummingbird wings have been shown to have superior lift-to-torque ratios over wings consisting of an attached leading-edge vortex as their governing aerodynamic mechanism (i.e. insects [39–44]) due to the development of a leading-edge suction force (${\bar{C}}_{L}/{\bar{C}}_{Q}>\cot\alpha$ [30,37,45]). Here, the P10 wing showed similar values of lift-to-torque ratios throughout the spectrum of angles of attack as that of the complete wing, outperforming the geometrical prediction for *α*>10° (figure 3*c*). Surprisingly, despite the substantial gaps in the P1-4 and SP6 wings, for *α*>15°, these wings also outperformed wings consisting of an attached leading-edge vortex. The higher angle of attack at which they succeeded in doing so, relative to the complete wing, serves as an indication of the effects of the large gaps in delaying the formation of a leading-edge vorticity structure. For *α*>20°, lift-to-torque ratios converged at the different moult stages and are analogous to cot⁡α, suggesting the presence of a leading-edge bubble over the wing throughout the moult sequence (figure 3*c*) [30].

### 3.2. Flow field measurements

**Figure 4.**
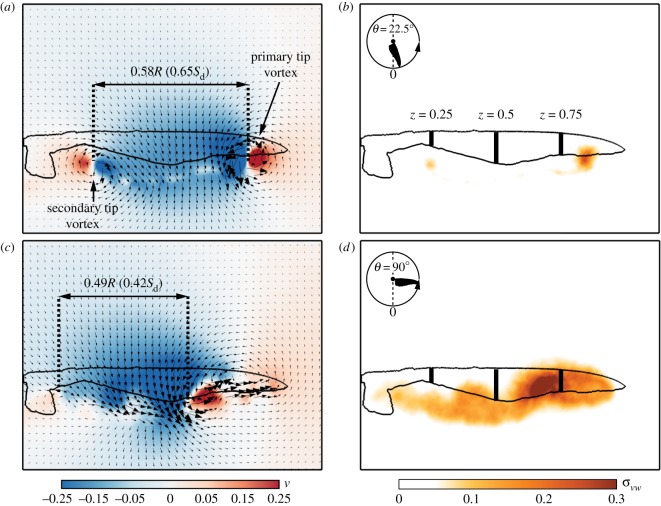
P1-4 near-wake measurements at *α*=30° and at (*a*,*b*) *θ*=22.5°; (*c*,*d*) *θ*=90°. Panels (*a*,*c*) depict the flow field with a background palette denoting *v*. Panels (*b*,*d*) represent the flow's fluctuation levels.

**Figure 5.**
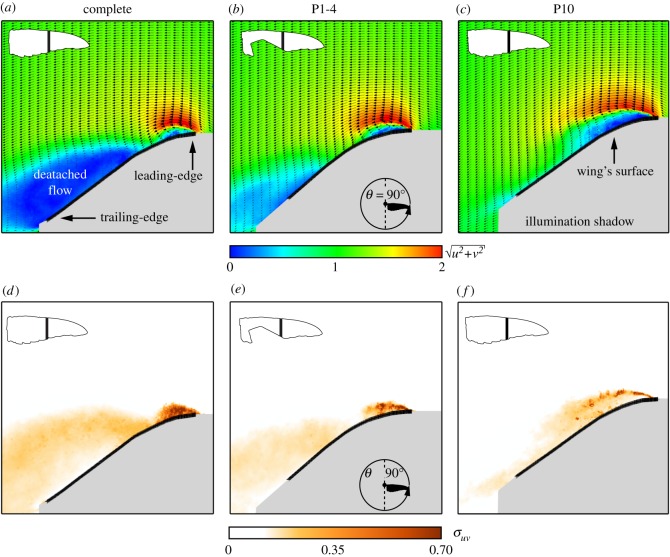
Flow fields (*a*–*c*) at *α*=30° and their corresponding flow fluctuation values (*d*–*f*) at mid-stroke over the medial span station (*θ*=90° and *z*=0.5) at different stages of moult: (*a*,*d*) complete wing; (*b*,*e*) P1-4 wing; (*c*,*f*) P10 wing. The first row's colour scheme describes the in-plane velocity magnitude, $\sqrt{u^{2}+v^{2}}$. The solid black line depicts the wing position and the shaded grey areas represent the illumination shadow region behind it.

As shown above, aerodynamic characteristics may vary considerably during moult. The following results (figures 4 and 5) are provided in order to represent the relationships between the flow field of the different moult stages and the wing aerodynamic characteristics. In light of the large reduction in lift capacity at the early stages of the moult cycle, when wing area is the smallest, we present near-wake measurements over a wing with missing P1-4 in figure 4. These measurements present the development of the tip vortex throughout the downstroke along with the effective disc area created over rotating or flapping wings and are characterized by negative vertical velocity values associated with lift. To enable a comparison to the complete wing, the effective disc area is provided in relation to the wing disc (*S*_*d*_=*π*[(*R*+*Rη*)^2^−(*Rη*)^2^] [30]).

Apart from the well-established primary tip vortex which formed at the wing tip at the early stages of the downstroke, a smaller, clockwise, secondary vortex appeared in the middle of the gap, effectively acting as a tip (*θ*=22.5°, figure 4*a*,*b*). The effective disc area is enclosed between the two vortices (primary and secondary) over the medial part of the wing, covering 65% of the wing disc area (figure 4*a*). At mid-stroke, the tip vortex is fully detached and fluctuating flow progressed from tip to root (figure 4*c*,*d*). The effective disc area migrated up the span and eventually covered 42% of the wing disc area. Overall, the missing surface of the wing induced a 16% reduction in the effective disc area compared to the complete wing throughout the downstroke (fig. 5 in [30]). The reduction in effective disc area yielded a qualitative measure of the lift degradation as evident by the aerodynamic loads obtained over the P1-4 wing (figure 3*a*).

To further understand the effect of moult on the main flow features, a comparison was made between the flow fields measured at mid-span and mid-stroke (*z*=0.5, *θ*=90°) over the complete, P1-4 and P10 wings (figure 5). The flow field over the complete wing shows two distinct flow features: an accelerated flow over the leading-edge covering about 0.3*c* (where *c* is the local chord length) and a large detached flow area forming behind the wing that is characterized by large values of flow fluctuations (figure 5*a*,*d*). As shown for the complete wing [30], these flow features developed until mid-stroke and remained similar in size and strength throughout the rest of the downstroke. Over the P1-4 wing (figure 5*b*), the accelerated area over the leading-edge is comparable in size and strength to that of the complete wing. The gap created by the missing feathers delayed the detachment of the flow, which continued to develop up until the end of the downstroke. The belated detachment might have been induced by the presence of the secondary tip vortex (figure 4), introducing enhanced mixing in the vicinity of the gap. This results in high momentum being inserted into the boundary layer, causing it to remain attached to the wing surface further along the downstroke.

The effects of the missing 10th primary flight feather are noticeable in figure 5*c*,*f*. At this stage, a large accelerated flow region developed over the leading-edge. Moreover, lower levels of flow fluctuations were measured. These flow fluctuations developed at the vicinity of the wing surface and are therefore associated with the boundary layer that developed along the chord. The increased effective leading-edge radius, though, only delayed the flow detachment over the span, fully detaching at *z*=0.75, identical to the *z*-value of the complete wing.

**Figure 6.**
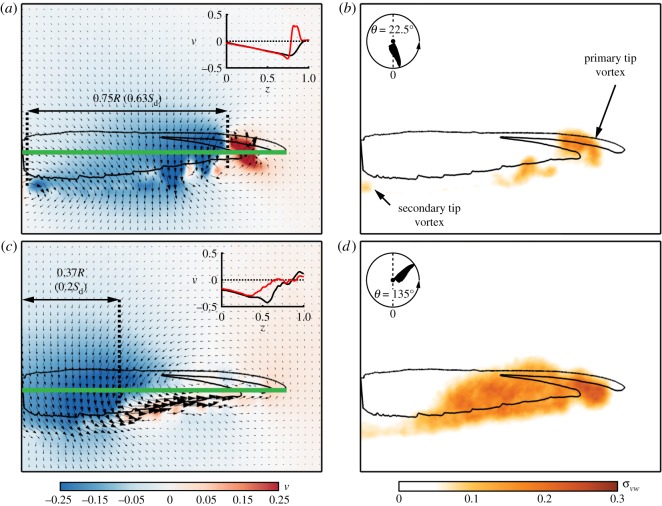
Near-wake measurements over a P89 wing configuration at *α*=30°; (*a*,*b*) *θ*=22.5°; (*c*,*d*) *θ*=135°. See figure 4 for panel description. In the insets, vertical velocity along the span (green line) is compared between the complete [30] (black line) and P89 (red line) wings.

We examined the significance of leading-edge and wing tip integrity by measuring the near-wake flow field of the P89 wing (figure 6). In this wing configuration, the wing is missing 11% of its surface area (table 1), and the gap is mostly found over the distal section of the wing (figure 2*e*). We found that the gap in the wing caused the tip vortex to break down even in the early stages of the downstroke (figure 6*a*,*b*), unlike the well-structured tip vortex that was present over the complete [30] and the P1-4 (figure 4*a*,*b*) wings. As a result, the effective disc area, stretching between the tip and root vortices, covered 0.63*S*_*d*_, a 19% reduction compared to the complete wing at this stage. Further down the stroke (figure 6*c*,*d*), the effective disc area covered an area of 0.2*S*_*d*_, a 40% decrease compared to that of the complete wing at this stage of the downstroke. As the effective disc area is associated with lift production, the large reduction in its size indicates a substantial aerodynamic impairment for this wing configuration. The distribution of the vertical velocity along the span of the P89 and complete wings further demonstrates the reduction in lift when the integrity of the wing tip is impaired (figure 6*a*,*c*). Although similar values of negative vertical velocity were measured within the effective disc area, beyond it, weaker negative and even positive vertical velocities were measured in the P89 wing compared to the complete wing, suggesting a further decrease in lift production by the wing.

## 4. Discussion

### 4.1. Flow field analysis

To investigate the effect of feather moult on the flow mechanisms over the *Calypte anna*'s wing and, subsequently, its connection to the wing's aerodynamic characteristics, below we present the spanwise vorticity field over the wing and the circulation at several span stations. A similar analysis was performed for the complete wing (fig. 6 in [30]).

#### 4.1.1. Early stages of moult

**Figure 7.**
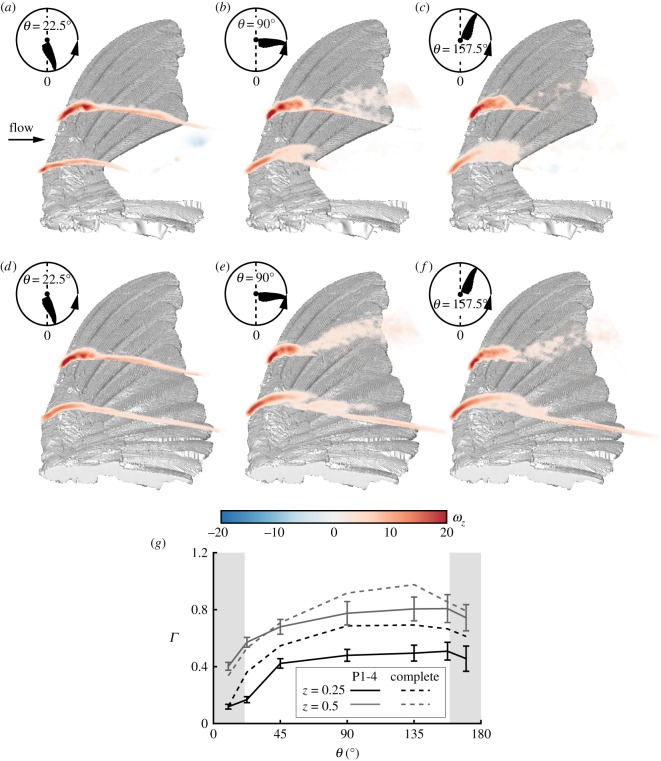
Spanwise vorticity fields at *α*=30° over the P1-4 wing at (*a*) *θ*=22.5°; (*b*) *θ*=90°; (*c*) *θ*=157.5°, and over the complete wing at (*d*) *θ*=22.5°; (*e*) *θ*=90°; (*f*) *θ*=157.5°. The vorticity fields are presented at the measured span stations *z*=0.25 and *z*=0.5. (*g*) Net circulation at the corresponding span stations over both wings. Grey areas are the acceleration and deceleration phases of the wing motion. Error bars represent the standard deviation of the P1-4 wing. For the standard deviation of the complete wing and wing kinematics, see [30].

We compared the dimensionless vorticity fields (${\hat{\omega }}_{z}$) at the early stages of the moult sequence, represented by the P1-4 wing, to those of the complete wing (figure 7). Despite the large gap in the wing geometry, just after the acceleration stage, where *θ*=22.5°, a small leading-edge bubble developed to a similar extent and strength as that of the complete wing (figure 7*a*,*d*; see also circulation values, *Γ*, in figure 7*g*). Lower values of circulation over the proximal span station (figure 7*g*) compared to the complete wing are associated with the clockwise starting vortex that is apparent at this stage (figure 7*a*). This vortex seems to remain in the vicinity of the wing's trailing-edge until *θ*=22.5°, somewhat longer than what was measured for the complete wing, shedding the vortex downstream at about *θ*=10°.

Later on, the gap in the wing disrupted the development of the leading-edge bubble at the proximal span station (figure 7*b*). This results in a weaker vorticity forming over the leading-edge, as also demonstrated by the lower circulation levels at this stage compared to that of the complete wing (figure 7*g*). Over the medial span station, where the leading-edge bubble is still of similar size and strength, lower values of positive circulation are associated with the development of the boundary layer (figure 7*b*,*e*). Maximal overall circulation is reached relatively late in the stroke (*θ*=157.5°) at both span stations over the P1-4 wing compared to *θ*=135° over the complete wing (figure 7*g*). These results clearly show that throughout most of the downstroke, the wing lift capacity is hampered. The integrated circulation along the downstroke over the P1-4 wing is 19% less than that of the complete wing, further supporting the lift force measurements that indicate a similar reduction in lift over the P1-4 wing (figure 3*a*).

#### 4.1.2. Late stages of moult

**Figure 8.**
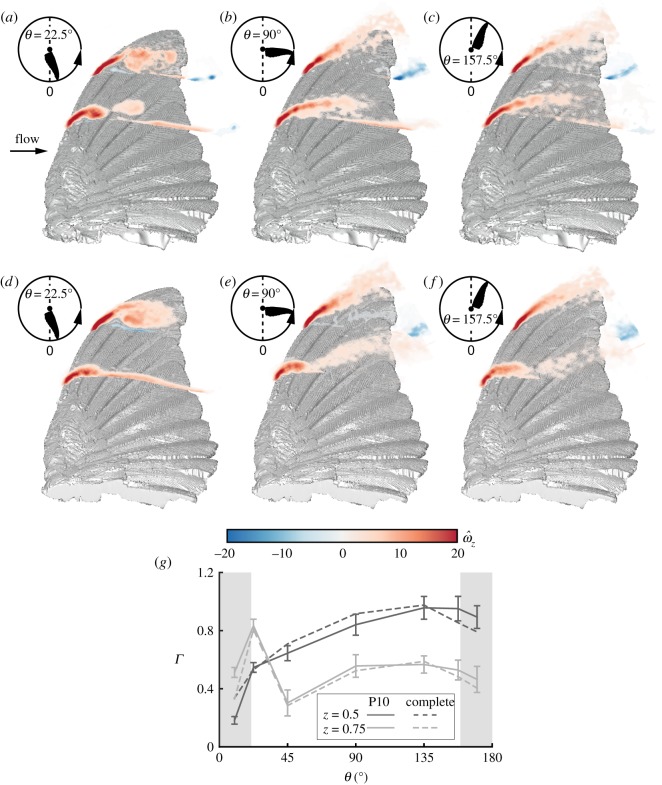
Spanwise vorticity fields at *α*=30° over the P10 wing at (*a*) *θ*=22.5°; (*b*) *θ*=90°; (*c*) *θ*=157.5°, and over the complete wing at (*d*) *θ*=22.5°; (*e*) *θ*=90°; (*f*) *θ*=157.5°. The vorticity fields are presented at the measured span stations *z*=0.5 and *z*=0.75. (*g*) Net circulation at the corresponding span stations over both wings. Grey areas are the acceleration and deceleration phases of the wing motion. Error bars represent the standard deviation of the circulation over the P10 wing. For the standard deviation of the complete wing and wing kinematics, see [30].

During the later stages of the moult sequence, represented by the P10 moult stage, the flight feathers forming the tip and the leading-edge of the wing are replaced. As suggested by the flow fields over mid-span at mid-stroke (figure 5*c*), a large leading-edge bubble developed over the P10 wing's leading-edge, covering about 0.6*c* and was maintained throughout the remainder of the downstroke (figure 8*a*–*c*). Interestingly, despite the seemingly large differences in flow field development (figure 5), the circulation measured over the P10 wing is almost identical to that which was measured over the complete wing throughout the entire downstroke (figure 8*g*), as was also suggested by the lift force measurements (figure 3*a*). Yet, at 22.5°<*θ*<135°, the circulation measured over the P10 wing is lower than that of the complete wing, showing an 8% decrease in circulation at mid-stroke. This finding agrees with the force measurements that were recorded for the P10 wing (figure 3*a*).

Regarding the flow field at the distal span station, the vorticity field was measured to be essentially the same as that of the complete wing as the flow fully detaches along the downstroke (figure 8). The circulation reaches its maximal value at this station just after the end of the acceleration stage (figure 8*g*). This is followed by an abrupt drop in circulation associated with flow detachment over the tip with the same magnitude as that recorded over the complete wing. Throughout the remainder of the downstroke, the overall circulation over the distal span station is as measured over the complete wing.

#### 4.1.3. The importance of the outer primaries: inferred from the flow over the P89 wing

Our ability to study complicated flows allowed us to further explore the biomechanics underpinning of the natural moult of hummingbirds by exploring the flow over a wing with a more extensive moult of primaries, in which two instead of only one feather that creates the wing tip were simultaneously shed. Our results demonstrate the utmost importance of the outer flight feathers and their integrity for lift production as indicated by the reduced effective disc area (figure 6). These results are in agreement with Chai [17], who reported the effect of gravely reduced hovering aerodynamic capacity when the wing tip of the ruby-throated hummingbird is cut off. Despite the ability to withstand larger morphological deficits to the wings during moult, a wing area loss of 10% at the wing tip resulted in failure to sustain hovering flight [17].

### 4.2. Wing performance during moult

**Figure 9.**
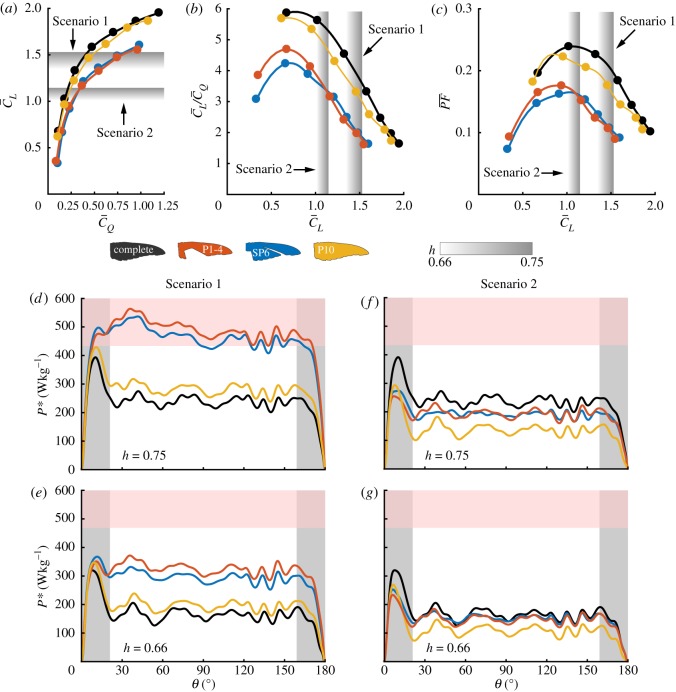
Downstroke performance during flight feather moult. (*a*) ${\bar{C}}_{L}$ versus ${\bar{C}}_{Q}$; (*b*) lift-to-torque due to drag ratio versus ${\bar{C}}_{L}$; (*c*) power factor versus ${\bar{C}}_{L}$; (*d*–*g*) time-accurate specific power (P*) during the downstroke at $\bar{\alpha }$ and at different moulting stages assuming: (*d*,*e*) no body mass reduction (Scenario 1); (*f*,*g*) 25% body mass reduction (Scenario 2). Rows are arranged with descending values of *h*: (*d*,*f*) *h*=0.75; (*e*,*g*) *h*=0.66. The shaded red region represents the lower downstroke specific power muscle limitations adopted from [46]. Grey areas are the acceleration and deceleration phases of the wing motion. For wing kinematics, see [30].

The downstroke aerodynamic characteristics of Anna's hummingbird wing during moult of its primary flight feathers are presented in figure 9. We distinguish two underlying factors to estimate the power requirements while hovering with a moulting wing, the bird's body mass (*m*_*b*_) and the downstroke contribution to the overall lift (and torque due to drag) produced by a wingbeat (downstroke and upstroke), *h* [30]. While numerous studies have discussed and evaluated these properties for hummingbirds during non-moulting periods of the year (*m*_*b*_=4.6 *g* [47]; 0.66≤*h*≤0.75 [48–51]), there is neither a comprehensive nor reliable database for these parameters during moult.

Regarding body mass, Chai [17] has reported that ruby-throated hummingbirds decrease their body mass by about 25% during moult at stages associated with wing area loss corresponding to the P1-4 and SP6 wings in our study. Similar results, albeit of a somewhat lesser degree of mass loss, were reported for rufous hummingbirds (*Selasphorus rufus* [28]). Epting [15] reported a 10% decrease in body mass of an individual black-chinned hummingbird (*Archilochus alexandri*) after breaking the distal tip of the 10th primary flight feather and different extents of body mass reduction at different moult stages on an individual Allen's hummingbird (*Selasphorus sasin*). However, as no equivalent data exist for Anna's hummingbirds, it is unknown if this species decreases its body mass during moult and to what extent. Furthermore, it remains to be seen if the birds reduce their body mass during moult regardless of their moult stage, or if they adjust their body mass in correspondence with the wing surface area during different stages of the moult sequence as was suggested by Epting [15]. If the latter is true, aerodynamic forces required for weight support may vary considerably during the entire moulting period because the hummingbird moult cycle involves reduction of wing area to different extents [22]. Moreover, hummingbird body mass varies significantly throughout the day, with fluctuations of up to 25% [25,26]. These uncertainties led us to suggest three scenarios which confine our aerodynamic performance analysis: (1) no body mass reduction during moult (*m*_*b*_=4.6 *g* [47]); (2) a 25% body mass reduction, corresponding to Chai's findings [17], which is assumed to be constant throughout the entire moulting period; and (3) a scenario similar to Scenario 2 but with a 10% body mass reduction at the P10 moult stage as inferred from Epting [15].

As presented in figure 3, the wing's capacity to generate lift (i.e. weight support) during the downstroke is reduced during moult. It is unknown, though, if the degradation in lift produced by the upstroke is proportional to that of the downstroke. For example, slits opened in the wing during the upstroke (between the S1 and P1 flight feathers [32]) might grow larger during moult, resulting in wing deformation and area reduction larger than the sum of the missing feathers, consequently affecting the lift produced during this phase. If so, *h* might change between periods in which the wing is complete and during moult (*h* will increase when the downstroke must account for more weight support). Furthermore, it is possible that each moult stage has its own value of *h*. To represent the variability of *h*, the aerodynamic performance estimation is presented for 0.66≤*h*≤0.75 in figure 9 and table 2 based on the range reported for the complete wing [48–51].

**Table 2.** Downstroke average specific power $\left({\bar{P}}^{*}\right)$ at $\bar{\alpha }$ for Scenarios 1–3. Complete wing values are in *W* *kg*^−1^; moult stage-specific powers are non-dimensional referenced to the corresponding complete wing value.

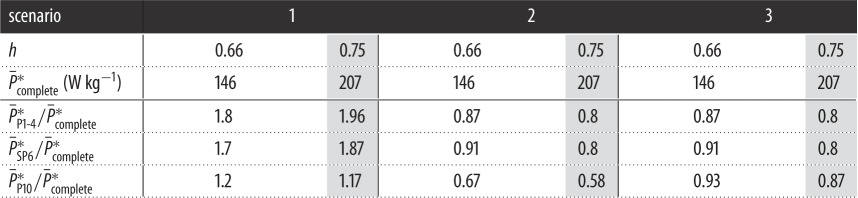

To provide sufficient lift during the downstroke, ${\bar{C}}_{L}=2h{\bar{C}}_{L_{b}}$ [30]. In the case of no body mass reduction during flight feather moult (Scenario 1), a mean value of ${\bar{C}}_{L}=1.4\pm 0.1$ (mean ± s.d due to *h* variation) is required from the downstroke in order to maintain hovering flight (figure 9*a*). At such conditions, Anna's hummingbird wing lift-to-torque ratio (figure 9*b*) and its ability to support a unit of weight ($\bar{PF}$ [30], figure 9*c*) decreased by about 33% and 50%, respectively, compared to that of the complete wing. At a moult stage where the P10 feather is missing, the wing performed similarly to the complete wing (figure 9*a*–*c*). Assuming that the bird reduces its body mass by 25% during the entire moult period (*m*_*b*_=3.45 *g*, Scenario 2), a more moderate downstroke average lift coefficient is required to sustain hovering flight, ${\bar{C}}_{L}=1.1\pm 0.1$ (figure 9*a*). This allowed the wing to perform at a more efficient working point, as can be inferred by the higher values of the lift-to-torque ratio and $\bar{PF}$ (figure 9*b*,*c*).

One of the dimensional parameters that may provide useful insights regarding Anna's hummingbird flight performance is the aerodynamic power required to sustain hovering flight. The downstroke time-accurate muscle mass-specific power (P*; see [30] for the mathematical expression) for Scenarios 1 and 2 at the tested range of *h* (at the corresponding downstroke average angles of attack, $\bar{\alpha }$; figure 9*a*) is presented in figure 9*d*–*g*. Previously reported flight muscles averaged power limitation for different species of hummingbirds [46] were modified to correspond to the pectoralis (downstroke flight muscles) specific power limit (see eqn. 4.6 in [30]). Doing so allowed us to offer a benchmark value for the power requirements estimated during moult for the different scenarios. Downstroke time-averaged specific power, defined as ${\bar{P}}^{*}=\int_{0}^{T}P^{*}/T$, is provided in table 2 for each scenario.

Following Scenario 1 and *h*=0.75, the relatively low efficiency induced a high aerodynamic power requirement for the P1-4 and SP6 wings—almost two times higher than that of the complete wing (table 2) and greater than the downstroke flight muscle power limit throughout most of the downstroke (figure 9*d*). On average, flapping at *h*=0.66 (figure 9*e*) decreased the cost of hovering by about 10% compared with that of the higher *h* factor, within a safety margin from the power limitation. Reducing body mass by 25% (Scenario 2) decreased the required power below that of the complete wing (figure 9*f*,*g*). Furthermore, the wing performed closer to its optimal $\bar{PF}$, making the specific power requirement less sensitive to variations in *h* (figure 9*c*, table 2). We note that the estimated wingbeat-specific power requirements (${\bar{P}}_{\mathrm{wb}}^{*}$; see [30] for the mathematical formulation) in this scenario for the P1-4 and SP6 wings are 69.4±6.7 W kg^−1^ and 70.7±4.7 W kg^−1^, respectively. These values are in very good agreement with Chai's estimation of moulting ruby-throated hummingbirds, with similar wing areas, assuming flight muscles mass is fixed during moult (72±6 W kg^−1^ and 69±3 W kg^−1^ [17]). These results support our experimental approach and provide a reliable estimation of the entire wingbeat performance during moult.

Interestingly, throughout Scenarios 1 and 2, the power requirements during the P10 moult stage are lower than the flight muscles limit. Reducing body mass according to Scenario 2 even reduced the specific power requirements by more than 33% compared to that of the complete wing (figure 9*f*,*g* and table 2). Therefore, mass reduction at the P10 moult stage is not a necessity in terms of aerodynamic performance in hovering. Applying a 10% body mass reduction at the P10 moult stage (Scenario 3) yielded power requirements which are similar to those of the complete wing and the rest of the moulting stages with a 25% body mass reduction (table 2). Under these circumstances, flight costs throughout the year vary by several per cent, as a result of properly adjusted body mass. Thus, one may infer that the extent of mass reduction during moult of the primary flight feathers is not constant and is determined by the basal (complete wing) flight cost.

## 5. Conclusion

Our findings provide inferences regarding the effect of moult on hummingbird wing aerodynamics. Our results suggest that depending on the moult stage, lift production can drop by more than 20% compared to the complete (non-moulting) wing. The largest effect of feather moult on wing performance was recorded when the medial flight feathers were missing, inducing large flow fluctuations that reduced the extent of the effective disc area. The feathers constituting the medial section of the wing, namely P5-7, are sequentially shed only after P1-4 are fully regrown [22], presumably as an outcome of natural selection to avoid gaps that may induce a substantial reduction in wing performance to an extent that may even cause aerodynamic failure.

The outer primary flight feathers are shed at the end of the moult sequence after the rest of the wing is renewed. Although the missing outer primary feathers at the later stages of the moult did not significantly affect lift production, the wing tip feathers were found to be essential for lift production. Flow field measurements over a wing with two outer primaries missing (P89) indicated a substantial drop of up to 40% in the effective disc area compared to that of the complete wing. This suggests that Anna's hummingbird wing performance is highly sensitive to morphological deficits at the wing tip. The importance of the integrity of the wing tip explains the increasing overlap between adjacent flight feathers towards the tip of the wing and the alteration of the moult sequence in Anna's hummingbirds [22], as well as in other hummingbird species [24], in which the outward moult sequence changes when reaching P9, minimizing the moult gap area at the wing tip.

Power and efficiency analyses are provided for a range of scenarios in which hummingbirds may operate during flight feather moult. These analyses suggest that Anna's hummingbirds may need to reduce their body mass during moult, at least at the early stages of the moult sequence, to withstand the limitations imposed by their flight muscles that must operate with reduced effective disc area. Furthermore, body mass variation may be governed by the interplay between biomechanical, physiological and behavioural factors and not only by power requirements during different periods of the year and even between different times of the day. To this end, we encourage further research on body mass variation and concomitant moult extent during the moulting period of free-ranging hummingbirds to enhance our understanding of the required weight support and aerodynamic and metabolic consequences of body mass and wing morphology at multiple time scales.

## Data accessibility

All data underlying this publication are available via Dryad (http://dx.doi.org/10.5061/dryad.mr556) [52].
